# Simvastatin Downregulates the SARS-CoV-2-Induced Inflammatory Response and Impairs Viral Infection Through Disruption of Lipid Rafts

**DOI:** 10.3389/fimmu.2022.820131

**Published:** 2022-02-18

**Authors:** Lívia Teixeira, Jairo R. Temerozo, Filipe S. Pereira-Dutra, André Costa Ferreira, Mayara Mattos, Barbara Simonson Gonçalves, Carolina Q. Sacramento, Lohanna Palhinha, Tamires Cunha-Fernandes, Suelen S. G. Dias, Vinicius Cardoso Soares, Ester A. Barreto, Daniella Cesar-Silva, Natalia Fintelman-Rodrigues, Camila R. R. Pão, Caroline S. de Freitas, Patrícia A. Reis, Eugenio D. Hottz, Fernando A. Bozza, Dumith C. Bou-Habib, Elvira M. Saraiva, Cecília J. G. de Almeida, João P. B. Viola, Thiago Moreno L. Souza, Patricia T. Bozza

**Affiliations:** ^1^Laboratory of Immunopharmacology, Oswaldo Cruz Institute, Oswaldo Cruz Foundation (FIOCRUZ), Rio de Janeiro, Brazil; ^2^Laboratory on Thymus Research, Oswaldo Cruz Institute, Oswaldo Cruz Foundation (FIOCRUZ), Rio de Janeiro, Brazil; ^3^National Institute for Science and Technology on Neuroimmunomodulation, Oswaldo Cruz Institute, Oswaldo Cruz Foundation (FIOCRUZ), Rio de Janeiro, Brazil; ^4^National Institute for Science and Technology on Innovation on Neglected Diseases (INCT/IDN), Center for Technological Development in Health (CDTS), Oswaldo Cruz Foundation (FIOCRUZ), Rio de Janeiro, Brazil; ^5^Preclinical Research Laboratory, Universidade Iguaçu (UNIG), Nova Iguaçu, Brazil; ^6^Program of Immunology and Inflammation, Federal University of Rio de Janeiro (UFRJ), Rio de Janeiro, Brazil; ^7^Biochemistry Department, Roberto Alcântara Gomes Biology Institute, State University of Rio de Janeiro, Rio de Janeiro, Brazil; ^8^Laboratory of Immunothrombosis, Department of Biochemistry, Federal University of Juiz de Fora (UFJF), Minas Gerais, Brazil; ^9^National Institute of Infectious Disease Evandro Chagas, Oswaldo Cruz Foundation, Rio de Janeiro, Brazil; ^10^D’Or Institute for Research and Education, Rio de Janeiro, Brazil; ^11^Laboratory of Immunobiology of Leishmaniasis, Department of Immunology, Paulo de Goes Institute of Microbiology, Federal University of Rio de Janeiro, Rio de Janeiro, Brazil; ^12^Program of Immunology and Tumor Biology, Brazilian National Cancer Institute (INCA), Rio de Janeiro, Brazil

**Keywords:** statin, COVID-19, inflammation, lipid rafts, SARS-CoV-2

## Abstract

Coronavirus disease 2019 (COVID-19) is currently a worldwide emergency caused by Severe Acute Respiratory Syndrome Coronavirus 2 (SARS-CoV-2). In observational clinical studies, statins have been identified as beneficial to hospitalized patients with COVID-19. However, experimental evidence of underlying statins protection against SARS-CoV-2 remains elusive. Here we reported for the first-time experimental evidence of the protective effects of simvastatin treatment both *in vitro* and *in vivo*. We found that treatment with simvastatin significantly reduced the viral replication and lung damage *in vivo*, delaying SARS-CoV-2-associated physiopathology and mortality in the K18-hACE2-transgenic mice model. Moreover, simvastatin also downregulated the inflammation triggered by SARS-CoV-2 infection in pulmonary tissue and in human neutrophils, peripheral blood monocytes, and lung epithelial Calu-3 cells *in vitro*, showing its potential to modulate the inflammatory response both at the site of infection and systemically. Additionally, we also observed that simvastatin affected the course of SARS-CoV-2 infection through displacing ACE2 on cell membrane lipid rafts. In conclusion, our results show that simvastatin exhibits early protective effects on SARS-CoV-2 infection by inhibiting virus cell entry and inflammatory cytokine production, through mechanisms at least in part dependent on lipid rafts disruption.

## Introduction

Coronavirus disease 2019 (COVID-19) is currently a worldwide emergency caused by Severe Acute Respiratory Syndrome Coronavirus 2 (SARS-CoV-2) (1). Although most individuals with SARS-CoV-2 infection develop only mild respiratory symptoms, during the most severe clinical manifestations of the disease, patients evolve to multiple organs and systems dysfunction, mimicking viral sepsis (2, 3). The susceptibility to developing severe COVID-19 is determined by various pre-existing conditions, such as old age, obesity, diabetes mellitus, and cardiovascular diseases (4, 5).

Since hypercholesterolemia is often observed in obesity, diabetes mellitus, and cardiovascular diseases, many people at high risk of developing severe COVID-19 use statins regularly to lower their cholesterol levels (6, 7). Statins are cholesterol-lowering drugs that inhibit hydroxyl-methylglutaryl coenzyme A (HMG-CoA) reductase, the rate-limiting enzyme in intracellular cholesterol synthesis (8). Simvastatin also decreases the cholesterol content of lipid rafts, thus displacing various proteins residing in these domains (9). Notwithstanding, several studies have shown that statins impair hyper inflammation and prevent thromboembolic events (8, 10, 11), making statins good candidates as adjuvant therapy for COVID-19 (12–14). Additionally, the pre-existing statin therapy has been associated with lower mortality and improved clinical parameters in COVID-19 compared to patients without statin therapy (2). Statin use prior to hospitalization reduces the risk of developing severe COVID-19 and accelerates patients’ recovery and hospital discharge (6, 7). Patients who remained on statins during hospitalization also exhibited a lower mortality rate compared to patients who stopped receiving statins after hospital admission (15). A recent updated meta-analysis including twenty-five cohorts and 147.824 patients also concluded that the use of statins is associated with a lower risk of mortality in COVID-19 patients (16).

Despite the great potential of simvastatin as an adjuvant treatment against COVID-19, the mechanisms underlying its suggested protection against SARS-CoV-2 infection are still poorly explored. Here we investigate the effects and mechanisms of statin-pretreatment in SARS-CoV-2 infection and inflammation. Using the COVID K18-hACE2-transgenic mouse model, we demonstrated that simvastatin reduced the viral replication and lung damage *in vivo*. We also provide evidence that the pleiotropic effects of simvastatin modulate the SARS-CoV-2-induced pro-inflammatory response. Moreover, our data suggest that simvastatin reduced viral replication by mechanisms at least in part dependent on lipid rafts disruption

## Materials and Methods

### Reagents

Simvastatin was purchased from Sigma-Aldrich (Cat#567020). Reconstitution was performed according to the manufacturer’s instructions.

### Study Approval

The *in vivo* studies were conducted according to the guidelines of Committee on the Use of Laboratory Animals of the Oswaldo Cruz Foundation (CEUA-FIOCRUZ) with license L003/21 and to the animal welfare guidelines of the Ethics Committee of Animal Experimentation from National Cancer Institute of Brazil (CEUA-INCA) with license 005/2021. Experimental procedures involving human cells from healthy donors were performed with samples obtained after written informed consent and were approved by the Institutional Review Board (IRB) of the Oswaldo Cruz Institute/Fiocruz (Rio de Janeiro, RJ, Brazil) under the number 49971421.8.0000.5248.

### Virus Strains and Growth Conditions

The SARS-CoV-2 D614G (GenBank #MT710714) and Gamma strains (#EPI_ISL_1060902) were originally isolated from nasopharyngeal swabs of a confirmed case from Rio de Janeiro/Brazil. after a single passage in cell culture, and virus expansion was performed in Vero E6 cells cultured in 150 cm^2^ flasks with high glucose DMEM plus 2% fetal bovine serum (FBS; HyClone) at 37°C in 5% CO_2_. Cytopathic effects were monitored daily and peaked 4 to 5 days after infection. According to WHO guidelines, all procedures involving handling infectious virus suspensions were carried out in a biosafety level 3 (BSL3) multiuser facility. Virus titers were determined as 50% of tissue culture infectious dose (TCID50/mL), and virus stocks were kept in -80°C ultralow freezers.

### Mice

K18-hACE2-transgenic mice (20–30 g) were obtained from the Oswaldo Cruz Foundation breeding colony. The animals were maintained with free access to food and water and kept at 25–28°C under a controlled 12 h light/dark cycle. Experiments were performed during the light phase of the cycle.

### Mice Treatment and Infections

For infection procedures, mice were anesthetized with 60 mg/kg of ketamine and 4 mg/kg of xylazine and inoculated intranasally with medium (Mock), or 10^5^ TCID of SARS-CoV-2 gamma strain in 25 µl of the medium. Treated groups received oral doses of 20 mg/kg of simvastatin 24 h and 1 h before infection (pretreatment). The same dose was given once daily for the subsequent days throughout the experiment. According to WHO guidelines, all procedures were performed in an Animal Biosafety Level 3 (ABSL-3) multiuser facility. Animals were monitored daily for eleven days for survival and body-weight analysis. The clinical score was determined by the observation of following signs: piloerection, curved trunk, alterations in gait, seizures, limb paralysis, coma, respiratory rate, skin color alterations, heart rate, lacrimation, palpebral closure, decreased grip strength, limb, abdominal and body tone and body temperature alterations. The clinical evaluation was based on a multifactorial SHIRPA protocol, with the modifications of Reis et al. (17).

### Bronchoalveolar Lavage and Lung Homogenates

To evaluate the inflammatory process induced by SARS-CoV-2 infection in the lung, mice were euthanized on day 6 after infection and bronchoalveolar lavage (BAL) from both lungs were harvested by washing the respiratory tract with 1 mL of cold PBS. After centrifugation of BAL (1500 rpm for 5 min), the pellets were used for total and differential leukocyte counts (diluted in Turk’s 2% acetic acid fluid) using a Neubauer chamber. Lactate dehydrogenase (LDH) activity was evaluated in centrifuged BAL supernatant to evaluate cell death (CytoTox96, Promega, USA). Differential cell counts were performed on cytocentrifuge preparations made using the Cytospin 3 (350 x g for 5 minutes at room temperature) and stained by the May-Grünwald-Giemsa method.

After BAL harvesting, lungs were perfused with 5 ml of PBS to remove the circulating blood and, then, collected, pottered, and homogenized in 750 µL of PBS containing the complete EDTA-free protease inhibitor cocktail (Roche Applied Science, Mannheim, Germany) for 30 sec, using an Ultra-Turrax Disperser T-10 basic IKA (Guangzhou, China). Homogenates were stored at −20°C for cytokine and chemokine measurements.

### Myeloperoxidase Activity

Myeloperoxidase activity was assayed spectrophotometrically using the method of Bradley et al. (18). Briefly, lung tissue sample was homogenized in 0.5% hexadecyltrimethylammonium bromide (HTAB - Sigma Chemical Co, St Louis, MO) in 50 mmol/L potassium phosphate buffer, pH 6.0. For each 50 mg of tissue, 100 μL of HTAB buffer was used. Suspensions were then centrifuged at 40,000g for 30 minutes and the resulting supernatant further assayed. Next, in 96-well plates, 50 μL of the supernatant was added together with 50 μL of HTAB plus 50 μL of orthodianisidine (0.167 mg/mL; Sigma Chemical) for 30 min at 37°C, and then 50 μL of H_2_O_2_ (0.0005%, Sigma Chemical) was added to each well. After 10 min, the samples underwent analysis in a spectrophotometer (460 nm), and the result was adjusted by tissue mg.

### Histological Procedure

Histological features related to the injury caused by SARS-CoV-2 infection were analyzed in the lungs of K18-hACE2 mice. Inflammatory and vascular infiltrates and evidence of cell degeneration was evaluated to characterize the level of the tissue damage. The collected material was fixed with formaldehyde (4%), dehydrated and embedded in paraffin to the obtention of tissue slices using a microtome. The slices were stained with hematoxylin and eosin and scanned for analysis using the Pannoramic Viewer program (3DHISTECH Ltd., Budapest, Hungary).

### Determination of Viral RNA Levels

Total RNA was extracted from lung homogenates using QIAamp Viral RNA (Qiagen^®^), according to manufacturer’s instructions. Quantitative RT-PCR was performed using GoTaq^®^ 1-Step RT-qPCR System (Promega) in a StepOne™ Real-Time PCR System (Thermo Fisher Scientific). Amplifications were carried out in 15 µL reaction mixtures containing 2× reaction mix buffer, 50 µM of each primer, 10 µM of the probe, and 5 µL of RNA template. Primers, probes, and cycling conditions recommended by the Centers for Disease Control and Prevention (CDC) protocol were used to detect the SARS-CoV-2 (CDC 2020). Amplification of the housekeeping gene HPRT1 (Mm03024075_m1, Thermo Fisher Scientific) was used as a reference for the number of cells used. The Ct values for this target were compared to those obtained with different cell quantities (10^7^ to 10^2^), for calibration.

### Cells and Virus and Reagents

African green monkey kidney cells (Vero, subtype E6) and the human lung epithelial cell lines (Calu-3) were expanded in high glucose DMEM with 100 U/mL penicillin and 100 μg/mL streptomycin (Pen/Strep; Gibco) and supplemented with 10% FBS. Cells were maintained at 37°C in a humidified atmosphere with 5% CO_2_.

PBMCs and neutrophils were isolated by density gradient centrifugation (Ficoll-Paque, GE Healthcare) from buffy-coat preparations of blood from healthy donors. Monocytes were isolated from PBMCs by plastic adherence. Briefly, PBMCs (2x10^6^ cells) were plated onto 48-well plates (NalgeNunc) in RPMI-1640 with 5% inactivated male human AB serum (Merck) for 2 h. Non-adherent cells were washed out, and the remaining monocytes were maintained in DMEM (low glucose) with 5% human serum (Merck), 100 U/mL penicillin, and 100 μg/mL streptomycin (Pen/Strep; Gibco). The purity of human monocytes was higher than 90%, as determined by flow cytometric analysis (FACScan; Becton Dickinson) using anti-CD3 (BD Biosciences) and anti-CD14 (Southern Biotech) monoclonal antibodies. Neutrophils were isolated after two hypotonic lysis of erythrocytes, washed with PBS, and resuspended in RPMI.

### NETosis and Measurement of Neutrophil-Derived Inflammatory Mediators

Neutrophils (2x10^5^ cells/well) were treated with simvastatin (10 μM) or medium for 30min at 37°C in RPMI, without serum. Then, cells were stimulated with PMA (100 nM) or inactivated SARS-CoV-2 (MOI 0.1) for 3 h, and the supernatants containing NETs were collected and centrifuged at 400 x g for 10 min to remove residual neutrophils. Quant-iT PicoGreen quantified the NETs. Neutrophils (2x10^5^/well) were treated with simvastatin (10 uM) or medium for 3 h at 37°C in RPMI, without serum. Next, cells were stimulated with PMA (100 nM), inactivated SARS-CoV-2 (MOI 0.1), and ROS production was evaluated using Dihydrorhodamine 123 (DHR) probe. (Excitation/Emission: 500/570 nm, SpectraMax Paradigm reader).

For microscopy analysis, neutrophils (1x10^5^/well) were seeded in Labteks (Nalge Nunc) and treated with simvastatin (10uM) for 30 minutes. Next, cells were stimulated with inactivated SARS-CoV-2 (MOI 0.1) for 3 h at 37°C and fixed with 4% formaldehyde. NETs were stained with DAPI and visualized by fluorescence using ZEISS Axio Imager D2 microscope. Cytokines and chemokines were quantified in the supernatants containing NETs by ELISA using commercial kits (R&D Systems), following the datasheet instructions.

### Cell Treatment, Viral Infections, and Viral Titration

Cells were treated with simvastatin at indicated doses for 24 h before infection. Then, the medium was removed, and infections were performed with SARS-CoV-2 D614G strain at a multiplicity of infection (MOI) of 0.01 (Vero E6 and monocytes) or 0.1 (Calu-3) in low (monocytes) or high (Vero E6 and Calu-3) glucose DMEM without serum. After the infection, the viral inoculum was removed, cells were incubated with complete fresh medium containing (or not) simvastatin and maintained at 37°C for an additional 24 h. Cells treated only after infections are referred to as post-treated cells.

For viral titration, the plaque-forming assay was performed in monolayers of Vero E6 cells seeded in 96-well plates (2 x 10^4^ cell/well). Cell monolayers were infected with serial dilutions of the supernatants containing SARS-CoV-2 for 1 h at 37°C. The cells were overlaid with high glucose DMEM containing 2% FBS and 2.4% carboxymethylcellulose (CMC/DMEM high glucose semisolid medium). After 3 days, the cells were fixed with 10% formaldehyde, and the monolayers were stained with 0.04% crystal violet in 20% ethanol for 1 h. Viral titers were calculated by counting the plaques formed in at least three dilution replicates and expressed as plaque-forming unit per mL (PFU/mL).

### Viral Adsorption and Internalization Assay

Monolayers of Calu-3 cells (3x 10^5^ cells/well in a 48-well plate) were treated with 25 µM simvastatin for 24 h. Thereafter, the medium was removed, and cells were exposed to SARS-CoV-2 at 0.1 MOI and incubated at 4°C for 1 h. For viral adsorption evaluation, following incubation time, cells were washed with cold PBS to remove the unabsorbed virus particles. Total RNA extraction and real-time PCR were performed as described above to measure the levels of viral adsorption. For internalization measurement, following incubation time, cells were washed with cold PBS and incubated at 37°C for 1 h. Then, cells were washed with PBS and treated with proteinase K (Invitrogen) for 45 min at 4°C to remove adsorbed but not internalized virus particles. After proteinase K inactivation with 2 mM PMSF in PBS-BSA, cells were pelleted in PBS containing 0.2% BSA. Following centrifugation of total RNA extraction, real-time PCR were performed as described above to measure the levels of viral internalization.

### Measurements of Inflammatory Mediators and Cell Death

IL-6, TNF, IFN-α, CCL2/MCP1, CCL5/RANTES, CXCL1/KC, CXCL8/IL-8, CXL10/IP10 levels were quantified in cell-free culture supernatants from infected or uninfected cells and in pulmonary extracts from infected mice by ELISA, following the manufacturer’s instructions (R&D Systems). Cell death was determined according to the activity of lactate dehydrogenase (LDH) in the culture supernatants and BAL using the CytoTox^®^ Kit (Promega, USA).

### SDS-PAGE and Western Blot

Cells were washed with ice-cold PBS and lysed in lysis buffer (1% Triton X-100, 2% SDS, 150 mM NaCl, 10 mM HEPES, 2 mM EDTA containing protease inhibitor cocktail (Roche, pH 8.0). After centrifugation at 13 000 g for 5 min, cell lysates were prepared in reducing and denaturing conditions and subjected to SDS-PAGE. Equal concentrations of proteins were fractionated by electrophoresis on 10% of acrylamide gels and were transferred onto a nitrocellulose membrane (Millipore, Billerica, MA, USA), followed by blocking of nonspecific binding sites in 5% nonfat milk in TBST (50 mM Tris-HCl - pH 7.4, 150 mM NaCl, 0.05% Tween 20) for 1 h at room temperature and blotted with primary antibodies in TBST overnight at 4°C. The following antibodies were used: anti-ACE2 (Proteintech-21115-1-AP), anti-TMPRSS2 (Proteintech-14437-1-AP), anti-GAPDH (Proteintech-60004-1-Ig), anti-CAV1 (Proteintech-16447-1-AP), and anti-FLOT1 (BD-610821). Proteins of interest were identified by incubating the membrane with IRDye^®^ LICOR or HRP-conjugated secondary antibodies in TBST, followed by detections by Supersignal Chemiluminescence (GE Healthcare) or by fluorescence imaging using the Odyssey system Odyssey^®^ (CLx Imaging System). Protein bands were quantified by densitometric image analysis using the ImageJ software.

### Immunofluorescence Staining

Calu-3 cells seeded in 48-well plates (2 x 10^4^ cell/well) with coverslips and the infections were performed with SARS-CoV-2 at MOI of 0.1 in high glucose DMEM without serum. After the infection, the viral inoculum was removed, cells were incubated with complete fresh medium containing (or not) simvastatin and maintained at 37°C for an additional 24 h. After 48h, the cells were fixed using 3.7% formaldehyde. Cells were rinsed three times with PBS containing 0.1 M CaCl2 and 1 M MgCl2 (PBS/CM) and then permeabilized with 0.1% Triton X-100 plus 1% BSA in PBS/CM for 10 min (PBS/CM/TB). Cells were stained with rabbit monoclonal antibody anti-ACE2 (Proteintech-21115-1-AP) at 1:200 dilution for overnight, followed by a rabbit anti-IgG-Dylight 488 (Invitrogen-35502) at 1:500 dilution for 1h. The coverslips were mounted in slides using an antifade mounting medium (VECTASHIELD^®^). Nuclear recognition was based on DAPI staining (1 μg/mL) for 5 min. Fluorescence was analyzed by fluorescence microscopy with an 60x objective lens (Olympus, Tokyo, Japan).

### Isolation of Detergent-Insoluble Membrane Domains

Cells grown in 75 cm^2^ flasks were washed three times with PBS saline buffer, scraped into 1.0 ml of lysis buffer [25 mM 2-morpholino-ethanesulfonic acid (MES), pH 6.5, 150 mM NaCl, 1% (v/v) Triton X-100 containing protease and phosphatase inhibitor cocktail (Roche)], and lysed using a Dounce tissue grinder, after moving the tight pestle up and down five times. An equal volume of 90% (w/v) sucrose in MBS (25 mM MES, 150 Mm NaCl, pH 6.5) was added to cell homogenates. The samples were then transferred to a 6 mL ultracentrifuge tube and overlaid with discontinuous sucrose gradients consisting of 35% (w/v) sucrose in MBS (2 ml) and 5% (w/v) sucrose in MBS (2 ml). The sucrose gradients were centrifuged at 100,000 g for 18 h at 4°C in a Beckman MLS50 rotor, and fractions of 0.5 ml were harvested from the top to the bottom of the tube. All steps were carried out at 4°C.

### Statistics

Statistics were performed using GraphPad Prism software version 8. Student’s T-test was used to compare differences between two groups. One-way analysis of variance (ANOVA) was used to compare differences among 3 groups following a normal (parametric) distribution with Dunnett’s *post-hoc* test was used to determine significant differences between groups; *p < 0.05; **p < 0.01; and ***p < 0.001.

## Results

### Simvastatin Treatment Reduced Virus Replication, Lung Damage and Delayed Pathophysiology of SARS-CoV-2-Infection in Mice

**Figure 1:**
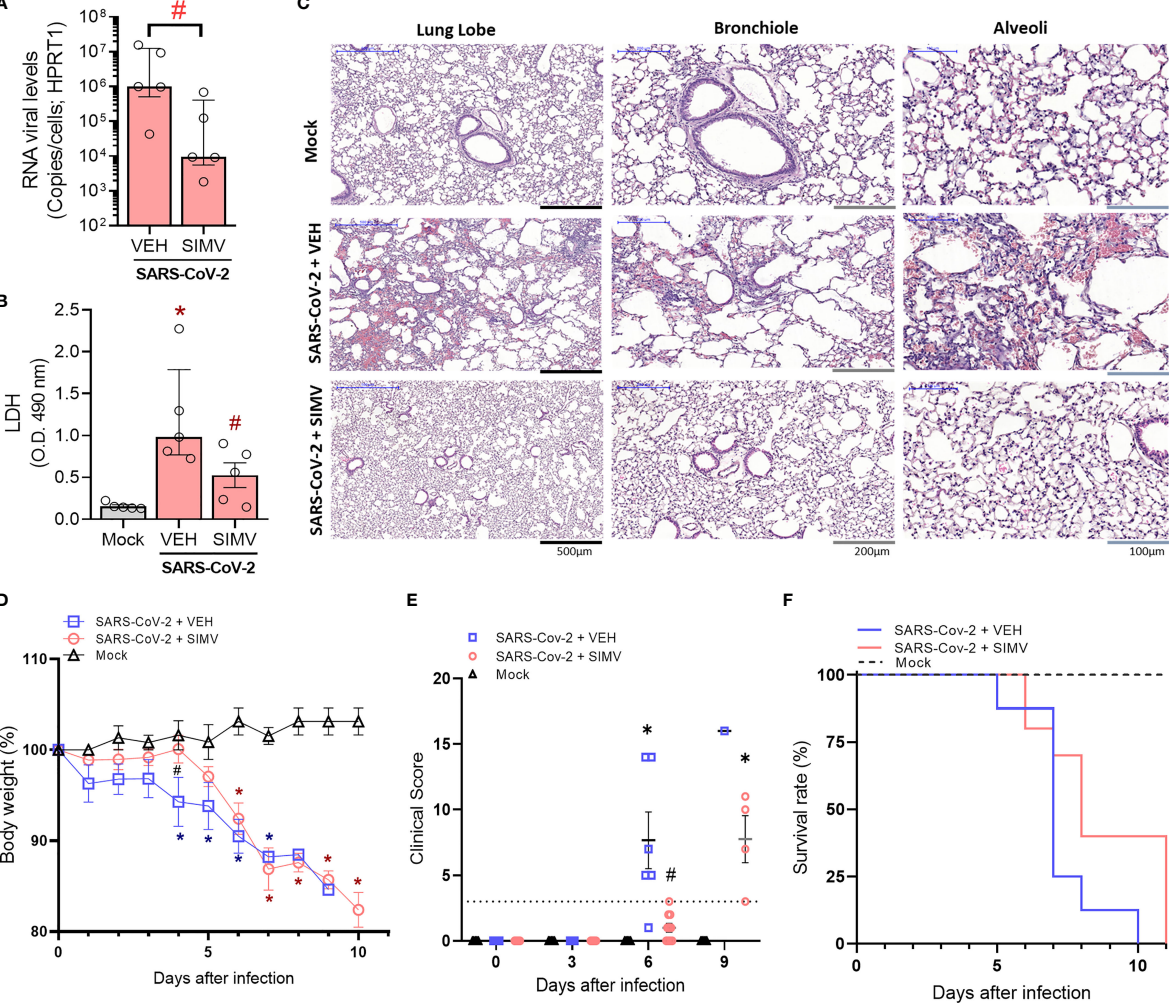
Effects of simvastatin in physiopathology of SARS-CoV-2 infection in K18-hACE2-transgenic mice. **(A–C)** K18-hACE2-transgenic mice were initially pretreated with 20 mg/kg of simvastatin 24h and 1h before infection by SARS-CoV-2 gamma strain. After infection, these animals continued to receive treatment daily with 20 mg/kg of simvastatin for six days post-infection. **(A)** SARS-CoV-2 RNA levels were measured in lungs from mice infected with SARS-CoV-2 treated with Vehicle (Veh) or simvastatin (SimV). **(B)** Lung damage was evaluated by measuring LDH release in the bronchoalveolar lavage (BAL) from mice infected with SARS-CoV-2 treated with Vehicle (Veh) or simvastatin (SimV). **(C)** Microphotographs for histology of lung lobe, bronchiole, and alveoli samples from mice infected with SARS-CoV-2 treated with Vehicle (Veh) or simvastatin (SimV). Data are expressed as median with interquartile range; Experiments were performed with 5 mice/group. **(D, E)** K18-hACE2-transgenic mice were initially pre-treated with 20 mg/kg of simvastatin 24h and 1h before infection by SARS-CoV-2 gamma strain. After infection, these animals continued to receive treatment daily with 20 mg/kg of simvastatin for eleven days post-infection. Weight variation **(D)**, clinical score **(E)**, and Survival **(F)** were assessed during all days after infection. Weight variation and clinical score data are expressed as means ± SD; Teste t de Student or One-way ANOVA with Dunnett’s *post-hoc* test, as recommended. *p < 0.05 in comparison to mock, #p < 0.05 comparison to infected untreated (Vehicle - Veh). Survival was statistically assessed by Log-rank (Mantel-Cox) test. (Mock n=5, SARS-CoV-2 + VEH n =8, SARS-CoV-2 + SIMV n = 10).

K18-hACE2-transgenic mice mimic many of the clinical features of COVID-19, making it one of the best animal models currently available (19, 20). As a proof-of-principle of the protective role of simvastatin for SARS-CoV-2 infection, K18-hACE2-transgenic mice were initially treated 24 h and 1 h before SARS-CoV-2 gamma strain infection with 20 mg/kg/day, a dose consistent with sepsis and malaria pre-clinical studies (17, 21). After infection, these animals continued to receive the same dose daily throughout the experiment period. Viral load measurements performed on the sixth day post-infection revealed a lower number of viral genome copies (**Figure 1A**) in the lungs of animals in the simvastatin group alongside lower tissue damage inferred through LDH levels BAL (**Figure 1B**). At this point, the histological analysis of lung tissue demonstrated that SARS-CoV-2 infection-induced diffuse alveolar damage (DAD) with leukocyte infiltration, epithelial denudation, submucosal congestion, edema, and hemorrhage (**Figure 1C**). Despite simvastatin not completely reversed the DAD and edema in SARS-CoV-2 infected mice, the treatment led to an important decrease in tissue hemorrhage and inflammation. Simvastatin pretreatment confers resistance in the early stages of disease onset but does not alter its late outcome (**Figures 1D–F**). Simvastatin delayed the weight loss (**Figure 1D**) and other parameters of the disease measured by clinical score (**Figure 1E**). As a result of the delay in the pathophysiology of infection, we identified that simvastatin led to an increase in survival time compared to the vehicle group (**Figure 1F**). However, simvastatin treatment failed to inhibit mortality at later time points.

### Simvastatin Impaired SARS-CoV-2- Induced Pro-Inflammatory Response in Murine Lung

**Figure 2:**
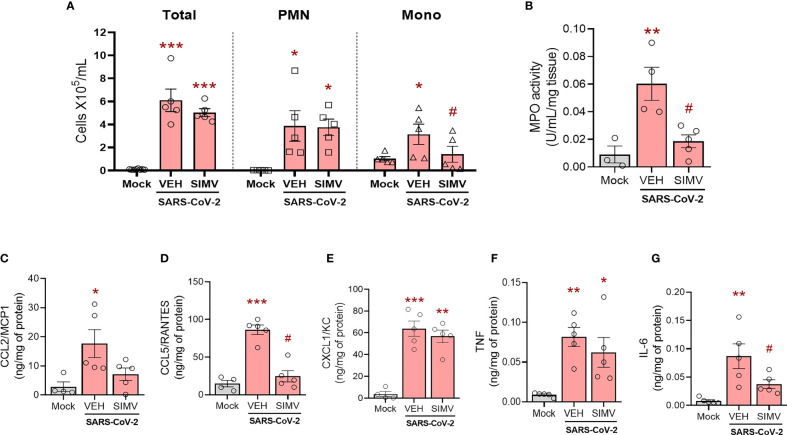
Effects of simvastatin in inflammatory induced by SARS-CoV-2 in murine lung. K18-hACE2-transgenic mice was initially pre-treated with 20 mg/kg of simvastatin 24h and 1h before infection by SARS-CoV-2 gamma strain. After infection, these animals continued to receive treatment daily with 20 mg/kg of simvastatin for six days post-infection. **(A)** Total and differential cell counts in bronchoalveolar lavage (BAL) were represented as number of differential cell counts. Total: leukocytes total, Mono: mononuclear leukocytes (Mono), PMN: polymorphonuclear leukocytes. **(B)** Myeloperoxidase (MPO) activity. The MPO activity in lung tissues were measured by the enzymatic assay. The level of CCL2/MCP1 **(C)**, CCL5/RANTES **(D)**, CXCL1/KC **(E)**, TNF **(F)**, and IL-6 **(G)** were measured by ELISA. Data are expressed as means ± SEM; One-way ANOVA with Dunnett’s *post-hoc* test. *p < 0.05, **p < 0.01, ***p < 0.01 in comparison to mock, #p < 0.05 comparison to infected untreated (Vehicle - Veh). Experiments were performed with 3-5 mice/group.

Overwhelming inflammation response is a pathological hallmark of COVID-19 disease, strongly associated with lung damage. Thus, we evaluated the effects of simvastatin on lung inflammation induced by SARS-CoV-2 infection. We observed that pretreatment with simvastatin failed to inhibit total leukocyte accumulation at the BAL on the sixth day of infection (**Figure 2A**). Simvastatin-treated animals presented a significant reduction in the number of mononuclear leukocytes (Mono) and a predominance of polymorphonuclear leukocytes (PMN) (**Figure 2A**). This phenomenon seems to be strongly associated with modifications of the main chemokines involved in leukocyte recruitment. We observed that simvastatin-treated group have an impaired the level of CCL2/MCP1 (**Figure 2C**) and CCL5/RANTES (**Figure 2D**), but not of level CXCL1/KC (**Figure 2E**) induced by SARS-CoV-2 infection. Of note, although simvastatin failed to inhibit neutrophil recruitment to the BAL, we observed a significant reduction in myeloperoxidase (MPO) detected in the lungs of simvastatin treated animals indicative of decreased accumulation of neutrophils in the lungs (**Figure 2B**). The pretreatment with simvastatin also significantly reduced the level of IL-6, but not TNF levels (**Figures 2F–G**), induced by SARS-CoV-2 infection in the lung tissue. Together, these data suggest that anti-inflammatory effects of simvastatin are also important components in reducing the pulmonary damage induced by SARS-CoV-2 infection.

### Simvastatin Reduces SARS-CoV-2- Induced Pro-Inflammatory Response in Human Neutrophils

**Figure 3:**
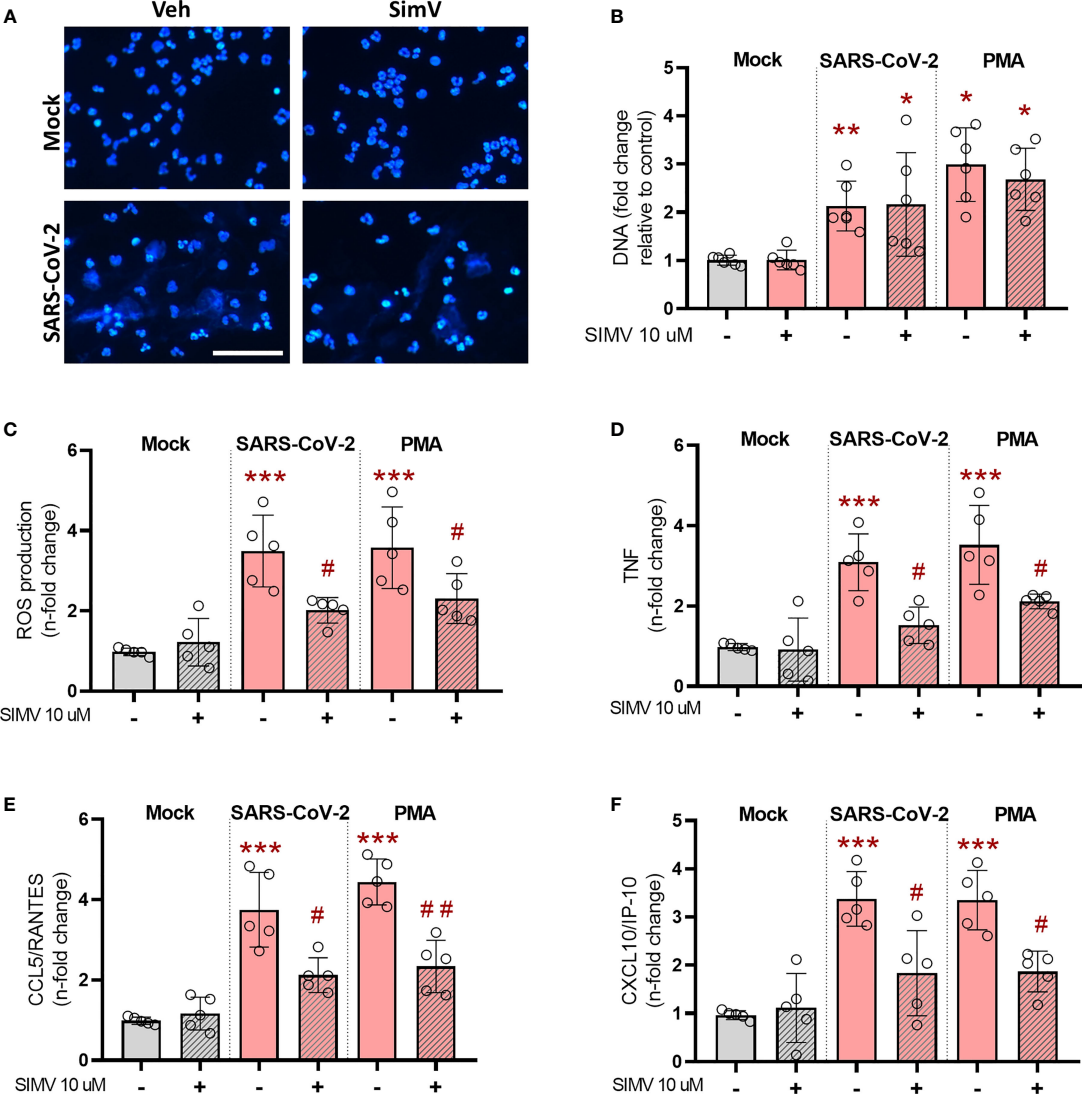
Effects of simvastatin on human neutrophils exposed to SARS-CoV-2. Human neutrophils were treated with simvastatin (10 μM) for 1 h before exposure to inactivated-SARS-CoV-2 (MOI of 0.1 for 3 h) or to PMA (100 nM) for 3 h. **(A)** Fluorescence analyses of NET after inactivated-SARS-CoV-2 estimulantion (MOI of 0.1) for 3h. NETs were stained with DAPI. Scale bar: 50 μm **(B–F)** Supernatants were then collected and centrifuged to remove residual neutrophils, and **(B)** NETs were quantified by Quant-iT PicoGreen. **(C)** ROS production was evaluated by using DHR (Dihydrorhodamine 1, 2, 3) probe **(D–F)**. Cytokines and chemokines were quantified in the supernatants containing NETs by ELISA. Data are expressed as means ± SD; *, comparison to mock; #, comparison to infected untreated; One-way ANOVA with Dunnett’s *post-hoc* test; n=5-6.

Activated neutrophils play central role in inflammatory response during COVID-19 but also contribute to lung damage through the process of degranulation and NETosis (22, 23). Thus, our next step was to assess the effects of simvastatin on neutrophils *in vitro*. Neutrophils exposed to inactivated SARS-CoV-2 virions increase NET formation (**Figures 3A, B**). Simvastatin pretreatment did not prevent NETosis induced by inactivated SARS-CoV-2 virions or PMA, a well-characterized agonist for NET formation (**Figure 3B**) However, simvastatin significantly reduced the oxidative stress measured by oxidation of DHR probe (**Figure 3C**) and the levels of inflammatory mediators TNF (**Figure 3D**), CCL5/RANTES (**Figure 3E**) and CXCL10/IP-10 (**Figure 3F**) induced by SARS-CoV-2 or PMA treatment.

### Simvastatin Downregulates Inflammatory Response Triggered by SARS-CoV-2 Infection in Human Monocytes

**Figure 4:**
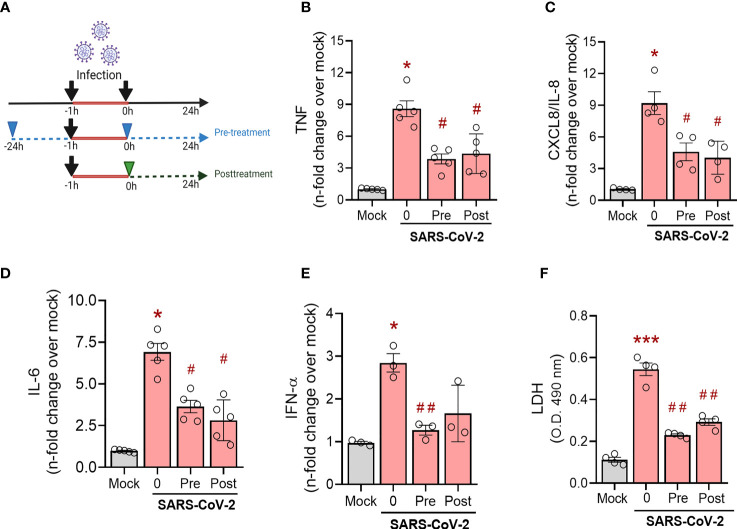
Effects of simvastatin on human monocytes infected by SARS-CoV-2. Monocytes from healthy donors were pre-treated or not with simvastatin (10 µM) then infected for 1 hour by SARS-CoV-2 h (MOI of 0.01). After removal of the virus inoculum, simvastatin was added again to the pre-treated group and added to infected cells as a posttreatment, at same concentration, for 24 hours **(A)** (The image was created with BioRender.com). The levels of TNF-α **(B)**, IL-8 **(C)**, IL-6 **(D)** and INF-α **(E)** were measured in culture supernatants by ELISA. **(F)** Cellular viability was analyzed by measuring LDH release in the supernatants of infected cells pre-treated or posttreated with simvastatin compared to the supernatant of uninfected cells or nontreated SARS-CoV-2-infected cells. Data are expressed as means ± SEM; #, comparison to mock; *, comparison to infected untreated; One-way ANOVA with Dunnett’s *post-hoc* test; n=3-6.

Next, we evaluated the ability of simvastatin to modulate the inflammatory response in SARS-CoV-2-infected monocytes, cells critically involved in COVID-19 pulmonary inflammation, using two treatment strategies, as shown in **Figure 4A**. First, we evaluated the simvastatin effect on pre-treated monocytes (treatment remains after infection period) and its therapeutic potential by post-treating these cells (**Figure 4A**). We found that either schedule of simvastatin treatment decreased the production of TNF, CXCL-8/IL-8, IL-6, and IFN-α (**Figures 4B–E**) induced by SARS-CoV-2 infection in human monocytes. Of note, both treatment strategies did not alter the levels of IL-1β, CCL2/MCP-1, CXCL10/IP-10, and IL-10 induced by SARS-CoV-2 infection (data not shown). Since SARS-CoV-2 infection is reported to induce monocyte death by pyroptosis (24), we also evaluated whether simvastatin treatment could reverse this phenomenon by measuring cytoplasmic LDH leak in culture supernatants. We observed that both pretreatment and posttreatment only with 10 µM of simvastatin prevented monocyte death induced by SARS-CoV-2 infection (**Figure 4F**).

### Simvastatin Reduces SARS-CoV-2- Induced Pro-Inflammatory Cytokine Release in Human Calu-3 Airway Epithelial Cells

**Figure 5:**
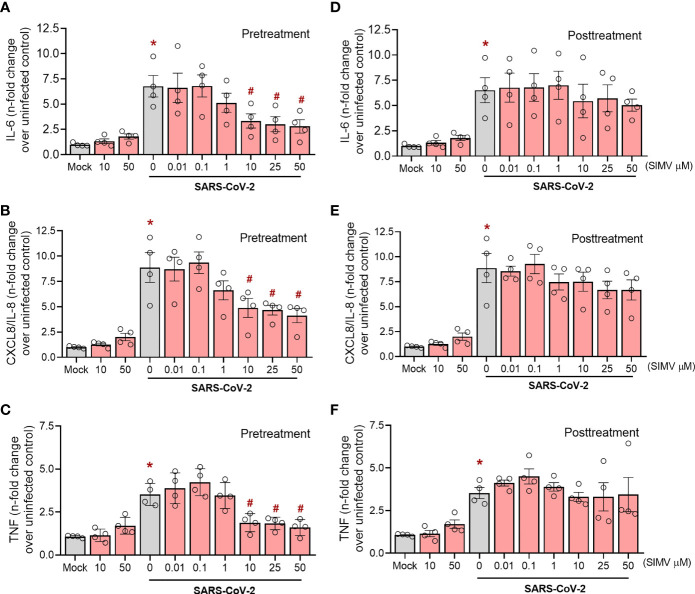
Effects of simvastatin on SARS-CoV-2 induced cytokine production in Calu-3. Calu-3 cells pre-treated or not with simvastatin were infected by SARS-CoV-2 with a MOI of 0.01 for 1 hours. After that, the viral inoculum was removed and simvastatin was replaced both in pretreatment and posttreatment group, for 48 hours. The levels of IL-6 **(A, D)**, IL-8 **(B, E)** and TNF-α **(C, F)** were measured in culture supernatants by ELISA. Data represent mean ± SD; #, comparison to Mock; *, comparison to infected untreated; One-way ANOVA with Dunnett’s *post-hoc* test. n=4.

To gain further insight on the effect of simvastatin, we also evaluated its impact on SARS-CoV-2 induced pro-inflammatory response in Calu-3 cells, a widely used human pulmonary epithelial cell to model susceptible cell types at the infectious site. Unlike monocytes, we observed a more prominent effect of pretreatment in the pro-inflammatory response induced by SARS-CoV-2 infection. Applying the same strategy for monocytes (**Figure 4A**), we found that pretreatment with simvastatin at 10 µM, 25 µM and 50 µM inhibited the release of IL-6, CXCL8/IL-8 and TNF by SARS-CoV-2-infected Calu-3 cells (**Figures 5A–C**). On the other hand, the posttreatment with simvastatin did not reduce the level of pro-inflammatory cytokines induced by SARS-CoV-2 infection in Calu-3 cells (**Figures 5D–F**). Taken together, these data strengthen the protective role of simvastatin against SARS-CoV-2 infection and suggest that simvastatin effects occur at the early stages of infection.

### Simvastatin Reduces SARS-CoV-2 Infection in the Human Airway Epithelial Calu-3 and in Vero E6 Cells

**Figure 6:**
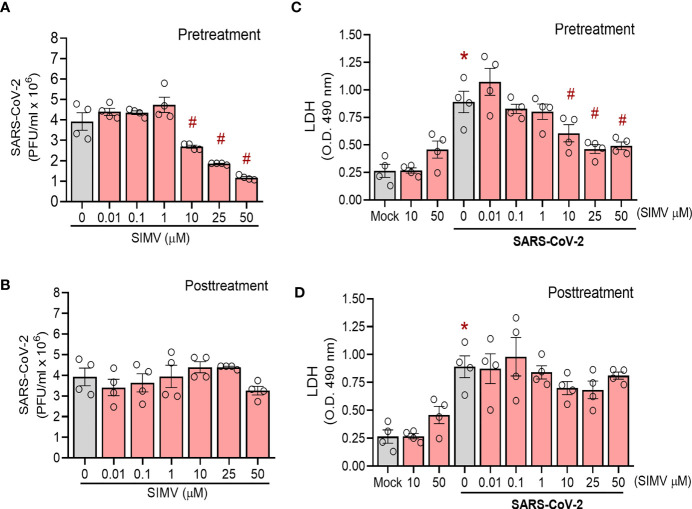
Effects of simvastatin on SARS-CoV-2 infection in Calu-3. Calu-3 were pre-treated with different concentrations of simvastatin (0.01, 0.1, 1, 10, 25 and 50 μM) for 24 hours before the infection with SARS-CoV-2 (MOI of 0.01 for 1h, in the absence of the drug). After infection, simvastatin was added again for additional 48 hours of incubation. Posttreatment refers to simvastatin treatment for 48 hour after infection only. Viral replication was determined in the cell-free supernatant by plaque assay. **(A, B)** Viral replication in cells **(A)** pre-treated or **(B)** posttreated only with simvastatin. **(C, D)** Cellular viability was analyzed by measuring LDH release in the supernatants of infected cells pre-treated **(C)** or posttreated **(D)** with simvastatin compared to the supernatant of uninfected cells or nontreated SARS-CoV-2-infected cells. Data are expressed a mean ± SD; #, comparison to Mock; *, comparison to infected untreated; One-way ANOVA with Dunnett`s *post-hoc* test. n=4.

Simvastatin’s capacity to lower cholesterol synthesis may alter plasma membrane composition, which is critical for viral infection. Based on this property, we evaluated the effect of simvastatin pre and posttreatment on SARS-CoV-2 infection in permissive cells. We observed that pretreatment with simvastatin reduced the viral load in Calu-3 cells in a dose-dependent manner, with an IC50 of 13.9 µM (**Figure 6A**). Along with the impairment of virus replication, simvastatin attenuated SARS-CoV-2-induced cell death as measured by LDH release (**Figure 6C**). On the other hand, the posttreatment with simvastatin failed to inhibit SARS-CoV-2 replication (**Figures 6B, D**). The same phenomenon was also observed in Vero E6 cells, a cell lineage highly permissive to SARS-CoV-2 infection. We observed that only pretreatment of Vero E6 cells with simvastatin reduced the infectivity of SARS-CoV-2 in a dose-dependent way (**Figure S1**).

### Simvastatin Inhibits SARS-CoV-2 Binding and Internalization Despite Upregulating ACE2 Expression in Calu-3 Cells

**Figure 7:**
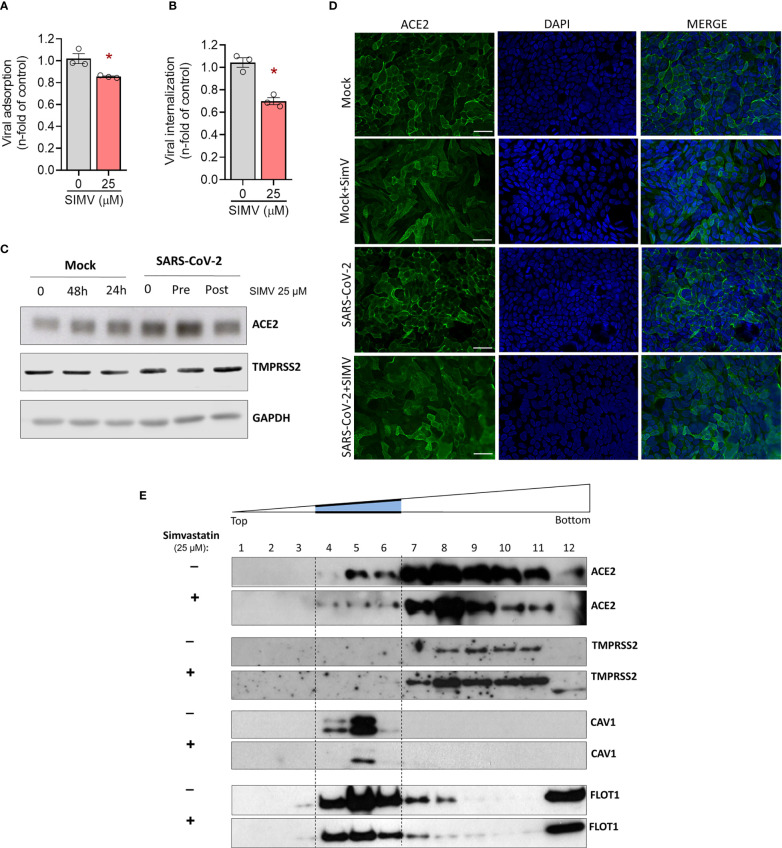
Simvastatin affects the expression level and location of ACE2, as well as adsorption and entry of SARS-CoV-2. Calu-3 were pre-treated or not with simvastatin 25 µM for 24h and then infected by SARS-CoV-2 with MOI of 0.1 at 4°C for 1h for viral adsorption assay **(A)** and at 4°C for 1h plus 1h at 37°C for viral internalization assay **(B)**. **(C)** Calu-3 pre-treated or not with simvastatin were infected by SARS-CoV-2 with a MOI of 0.01 for 1 hours. Thereafter, the inoculum was removed, and simvastatin were added again both in pretreatment and posttreatment group, for 24-hour incubation. Cell lysates were collected for the detection of ACE2 and TMPRSS2 by Western blotting. GAPDH levels were used for control of protein loading. **(D)** Immunofluorescence analyses of Calu-3 after SARS-CoV-2 infection with MOI of 0.01 for 48h. ACE2 was detected by indirect immunofluorescence (Green), nuclei were stained with DAPI (Blue). Scale bar: 20 µm. **(E)** Calu-3 cells treated or not for 24h with 25 mM simvastatin were lysed in cold Triton X-100-containing buffer and solubilized proteins were separated by ultracentrifugation on discontinuous sucrose density gradients. Detection of proteins was determined by Western blotting with specific antibodies. Caveolin-1 and flotillin-1 were used as markers of detergent-insoluble fractions. Data are expressed as mean ± SD; *, comparison to infected untreated, Paired t-test. n=3-4.

To further gain insight on the mechanism by which simvastatin impairs SARS-CoV-2 infection, we investigated whether this compound affects SARS-CoV-2 adsorption and internalization. Our data show that simvastatin significantly inhibits virus adsorption (**Figure 7A**) and virus entry (**Figure 7B**), which could contribute to further reduced virus production. To understand the mechanism of action of simvastatin on SARS-CoV-2 attachment and entry, we evaluated the level of expression of ACE2 and TMPRSS2, which SARS-CoV-2 explores for cell entry. Surprisingly, ACE2 expression was enhanced after 24 h and 48 h of simvastatin treatment (**Figure 7C**). Despite the enhancement of ACE2 expression by simvastatin pretreatment, SARS-CoV-2 binds to the cell surface less efficiently (**Figure 7A**) and virus internalization is reduced (**Figure 7B**). We also detected an increase of ACE2 expression level in Vero E6 cells after 24 h treatment with simvastatin and a further increase after SARS-CoV-2 infection compared to cells infected but not treated with simvastatin (**Figure S2**). On the other hand, the expression levels of TMPRSS2, the cellular serine protease that primes SARS-CoV-2 Spike protein for entry (25), remained unchanged in all conditions analyzed in both cell types (**Figure 7C** and **Supplementary Figure S2**). These findings suggest that, although simvastatin treatment increases the global expression of ACE2 in the cell, it somehow prevents the correct interaction of the viral spike protein with its receptor, probably due to the well-known effect of simvastatin in disrupting lipid rafts.

### Simvastatin Pretreatment Moves ACE2 Out of Lipid Rafts in Human Calu-3 Epithelial Lung Cells

To advance the understanding of the mechanisms by which simvastatin pretreatment impairs adsorption and internalization of SARS-CoV-2, we decided to study the impacts of this treatment also in the subcellular location of ACE2 (**Figure 7D**). In mock condition, ACE2 is localized primarily at the cell surface as expected for this cell line. The pretreatment with simvastatin induces a subcellular redistribution of ACE2. The intense concentration of receptors on the plasma membrane were no longer visible and green signals were instead seen as a diffuse labeling. Since statins directly interfere with the integrity of cholesterol microdomains in the plasma membrane, the receptors are likely to have diffused across the lipid bilayer and/or been internalized. To confirm whether the effect of simvastatin in SARS-CoV-2 is related to rafts disturbance, we also evaluated the location of ACE2 and TMPRSS2 in lipid rafts by protein solubilization in cold Triton X-100 buffer, followed by cell fractionation in discontinuous sucrose gradient. In control cells, a substantial amount of ACE2 co-fractionates with the raft marker caveolin-1 (Cav-1) and flotillin-1 (FLOT1) in fractions 4-6 (**Figure 7E**). In contrast, TMPRSS2 was confined to the detergent-soluble fractions 7-11. Treatment with simvastatin promoted the movement of ACE2 to the detergent-soluble fractions, while the localization of TMPRSS2 in the cellular membrane remained unchanged. Altogether, our data suggest that simvastatin inhibits SARS-CoV-2 binding and entry to the cell by disrupting the lipid environment of the plasma membrane raft structure, altering the location of ACE2, and thus, impeding the proper interaction with the virus.

## Discussion

Statins combine antiinflammatory, antithrombotic, and immunomodulatory effects that potentially meet the main demands of those affected by COVID-19 (12, 26). Several retrospective observational clinical studies have recently attested to the beneficial effects of previous and prolonged use of statins and their in-hospital introduction after SARS-CoV-2 infection on patients’ prognosis (7, 15, 27, 28). However, experimental evidence showing how the use of statins can affect the course of SARS-CoV-2 infection is scarce. We demonstrated that simvastatin reduced viral replication and production of inflammatory mediators induced by SARS-CoV-2 *in vivo* and *in vitro* infected cells. In addition, simvastatin delayed the weight loss and mortality induced by SARS-CoV-2 in ACE2 transgenic mice. However, simvastatin treatment failed to inhibit mortality at later time points.

As a sepsis-like phenomenon, the systemic cytokine storm contributes to airways and extrapulmonary organ damage (29). Our results suggest that the regulation of the inflammatory response triggered by SARS-CoV-2 in COVID-19 patients may be an important component in the resistance conferred to statin users as well as those who received statins in-hospital. In this sense, the antiinflammatory effect of statins is a well-characterized phenomenon in several infection models (10, 11, 30, 31). Moreover, statins downregulate pro-inflammatory response by inhibiting both NF-κB-mediated cytokine induction and NLRP3 inflammasome activation (13), essential components for the hyperinflammatory response triggered by SARS-CoV-2 infection (24, 25, 32). This antiinflammatory effect of statins may explain the delayed pathophysiology of COVID-19 and mortality, and lower recruitment of monocytes to the lung. Moreover, monocytic cells are a major cellular component of the pulmonary cell infiltrate, and macrophage activation syndrome (MAS)-like accounts for several features of COVID-19 pathogenesis (33). On the other hand, NETs released by SARS-CoV-2–activated neutrophils have also been associated with lung damage and promote lung epithelial cell death *in vitro* (23). These results unravel a possible detrimental role of NETs in the pathophysiology of COVID-19. In this context, we observed that simvastatin, although it decreased the pro-inflammatory and oxidative response to SARS-CoV-2 in human neutrophil, failed in reducing NET release. Furthermore, simvastatin’s protective and antiinflammatory effects appear to be primarily related to the early events of SARS-CoV-2 infection, both *in vitro* and *in vivo*. Remarkably, in human pulmonary epithelial cells, the 24 h before infection pretreatment and maintained thereafter with simvastatin downregulated the secretion of pro-inflammatory cytokines, whereas 1 h posttreatment was devoided of effect. Similarly, in peripheral blood monocytes, 24h pretreatment was more effective when compared to posttreatment only.

SARS-CoV-2 interacts with receptors embedded in the plasma membrane in specifically organized microdomains known as lipid rafts (34). Lipid rafts are cholesterol- and sphingolipids-enriched plasma membrane regions that have reduced fluidity and selectively incorporate or exclude proteins (35–37). Thus, lipid rafts can compartmentalize specific components and mediate important cell events as a signaling platform (38). Numerous enveloped viruses, including members of the *Coronaviridae* family (37), exploit lipid rafts in distinct steps of their life cycle, such as cell entry, trafficking, signaling, and budding of the cell (39). Data from our adsorption assay confirmed that treatment with simvastatin impairs virus-cell tethering and reduces the number of viral particles that reach the intracellular compartment. This result agrees that simvastatin disrupts the composition and properties of lipid rafts (37), which was proven to be essential for the interaction of SARS-CoV spike protein. Accordingly, recently it has been shown that the depletion of cholesterol in the plasma membrane and its abnormal redistribution into intracellular compartments slows down the kinetic of SARS-CoV-2 infection by suppresses SARS-CoV-2 S protein-mediated fusion, which inhibits virus replication (40, 41). Moreover, other raft-resident lipids such as sphingolipids and ceramides were seen as important in the mechanisms of fusion and virus entry into the cell, highlighting the importance of the integrity of lipid rafts in these mechanisms (42). In this context, the pharmacological redistribution of cholesterol and impaired lipid raft formation hampers the SARS-CoV-2 SARS-CoV-2 entry, and consequently the virus replication (34, 43–45). In line with the results obtained here with simvastatin pretreatment, other drugs that affect not only the synthesis but also the traffic of cholesterol through the endosomal pathway, such as itraconazole, fluoxetine and amitriptyline showed promising results as adjuvant therapy for COVID-19 (42, 46, 47).

Interestingly, as reported by our study and others (48–52), statins can increase the expression of ACE2, the central receptor for recognition and entry of SARS-CoV-2 into the host cell (53, 54). However, subcellular fractionation revealed that the ACE2 receptor is misplaced after simvastatin treatment, being reduced in lipid rafts despite its global enhancement. Therefore, these results suggest that simvastatin impairs the early stages of SARS-CoV-2 infection by disrupting lipid rafts and preventing the proper interaction of viral particles with ACE2 receptors. Similar results were obtained by depletion of plasma membrane cholesterol with methyl-β-cyclodextrin (MβCD), which disrupts lipid rafts and relocates ACE2 to a non-raft environment impacting SARS-CoV infectivity (55). ACE2 is a master regulator of the renin-angiotensin system (56) and has shown to be protective during SARS-CoV-2 infection by downregulating Ang II-mediated vascular permeability and acute lung injury (57, 58). The activity of ACE2 provokes a shift from Ang II toward Ag-(1-7) availability, which has been shown to reduce systemic inflammation in a phase-2 clinical trial of recombinant ACE2 (59). Thus, the elevated levels of ACE2 induced by simvastatin are not necessarily detrimental during SARS-CoV-2 infection.

In this work, we investigated whether simvastatin would have protective effects against COVID-19 *in vivo* and *in vitro* and shed light on mechanisms of action. However, it is crucial to highlight some limitations of this study. The dose of simvastatin used in mouse experiments was based on numerous studies that use between 10-40 mg/kg to evaluate the effects of simvastatin in different infection and sterile models of disease (17, 21, 49, 60–62). However, it is important to note that these doses are higher than that routinely applied in human clinical use, limited to a maximum of 80 mg per day (63, 64). As such, the doses used in this article in mice cannot be extrapolated to humans and future randomized clinical studies would be necessary to confirm the potential beneficial effects to humans. Nevertheless, our results suggest that simvastatin treatment may have adjuvant beneficial therapeutic effect on COVID-19 as for patients who already have been using the statins chronically.

**Figure 8:**
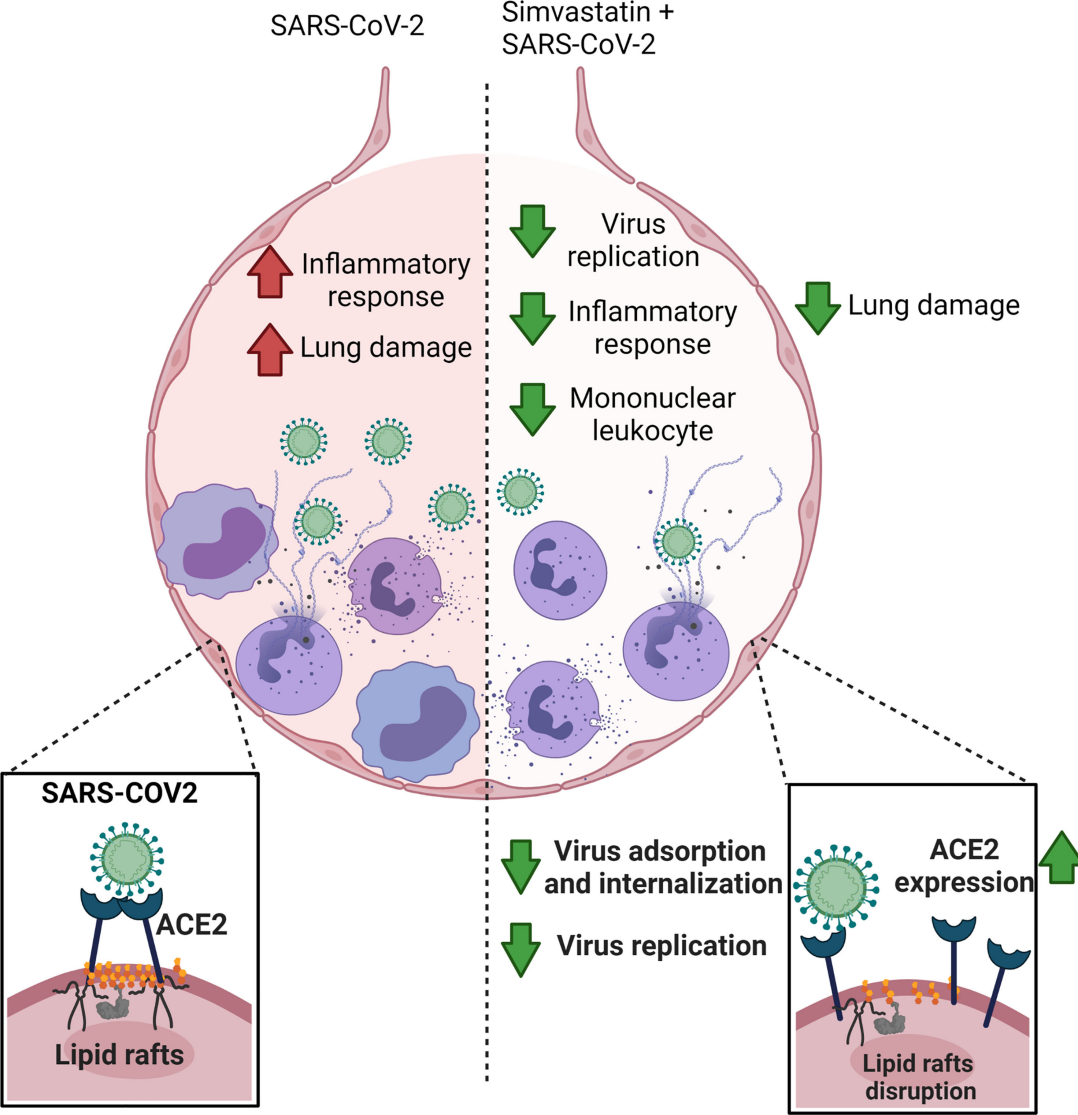
Simvastatin have prophylactic effect against COVID-19. Simvastatin pretreatment significantly reduced the viral replication and lung damage *in vivo*, delaying SARS-CoV-2-associated physiopathology and mortality in the K18-hACE2-transgenic mice model. *In vitro* models, Simvastatin treatment downregulated key pro-inflammatory cytokines triggered by SARS-CoV-2 infection in human neutrophils, peripheral blood monocytes and lung epithelial Calu-3 cells, showing the potential to modulate the inflammatory response both at the site of infection and systemically. Additionally, Simvastatin pretreatment affects the course of SARS-CoV-2 infection by inhibiting virus entry through mechanisms of ACE2 displacement of rafts. The image was created with BioRender.com.

In summary, our data showed that simvastatin is endowed with the ability to reduce the inflammatory response associated with SARS-CoV-2 infection both *in vivo* and *in vitro* (**Figure 8**). Our results suggest that simvastatin-induced modifications in cellular components are essential to decrease the inflammatory response related to COVID-19. Moreover, our data demonstrate that simvastatin disrupts lipid rafts relocating ACE2 to the non-raft membrane, which possibly hampers interaction of ACE2 and SARS-CoV-2, preventing infection (**Figure 8**). Thus, our data support the clinical observations of the protective effect of previous statins in conferring resistance to SARS-CoV-2 infection. However, the therapeutic potential of statins as adjuvant therapy against COVID-19 should be further evaluated in randomized controlled clinical trials.

## Data Availability Statement

The original contributions presented in the study are included in the article/Supplementary Material. Further inquiries can be directed to the corresponding author.

## Ethics Statement

The studies involving human participants were reviewed and approved by Institutional Review Board (IRB) of the Oswaldo Cruz Institute/Fiocruz (Rio de Janeiro, RJ, Brazil) under the number 49971421.8.0000.5248. The patients/participants provided their written informed consent to participate in this study. The animal study was reviewed and approved by Committee on the Use of Laboratory Animals of the Oswaldo Cruz Foundation (CEUA-FIOCRUZ, license L003/21), and to the animal welfare guidelines of the Ethics Committee of Animal Experimentation from National Cancer Institute of Brazil (CEUA-INCA) with license 005/2021.

## Author Contributions

LT and PB conceptualized the study. LT, JT, and FP-D were equally responsible for design and conducted the experiments, literature review, prepared the figures and tables. The manuscript was written by LT, JT, and FP-D, and edited by all authors. CS and NF-R performed virus isolation and titration. MM, AF, and CS performed *in vivo* treatment and infection. BSG, TC-F, SD, VS, EB, DC-S, LP, CP, PR, EH, and CF contributed to the *in vitro* data collection and analysis. PB, TS, DB-H, EMS, CA, FB, and JV contributed to study design and critically revised the article. All authors approved the submitted version.

## Funding

This study was supported by the Coordenação de Aperfeiçoamento de Pessoal de Nível Superior – Brasil (CAPES), by the Fundação de Amparo à Pesquisa do Estado do Rio de Janeiro (FAPERJ), by the Conselho Nacional de Desenvolvimento Científico e Tecnológico (CNPq) and Mercosur Structural Convergence Fund (FOCEM, Mercosur, grant number 03/11, to Laboratory of Thymus Research), and by Oswaldo Cruz Foundation/Fiocruz Inova Program. The funders had no role in study design, data collection and analysis, decision to publish, or preparation of the manuscript.

## Conflict of Interest

The authors declare that the research was conducted in the absence of any commercial or financial relationships that could be construed as a potential conflict of interest.

## Publisher’s Note

All claims expressed in this article are solely those of the authors and do not necessarily represent those of their affiliated organizations, or those of the publisher, the editors and the reviewers. Any product that may be evaluated in this article, or claim that may be made by its manufacturer, is not guaranteed or endorsed by the publisher.
