# Supplementary figures and images for: Simvastatin Downregulates the SARS-CoV-2-Induced Inflammatory Response and Impairs Viral Infection Through Disruption of Lipid Rafts
Source: Front Immunol. 2022 Feb 18;13:820131. doi: 10.3389/fimmu.2022.820131 (PMC8895251; doi:10.3389/fimmu.2022.820131)

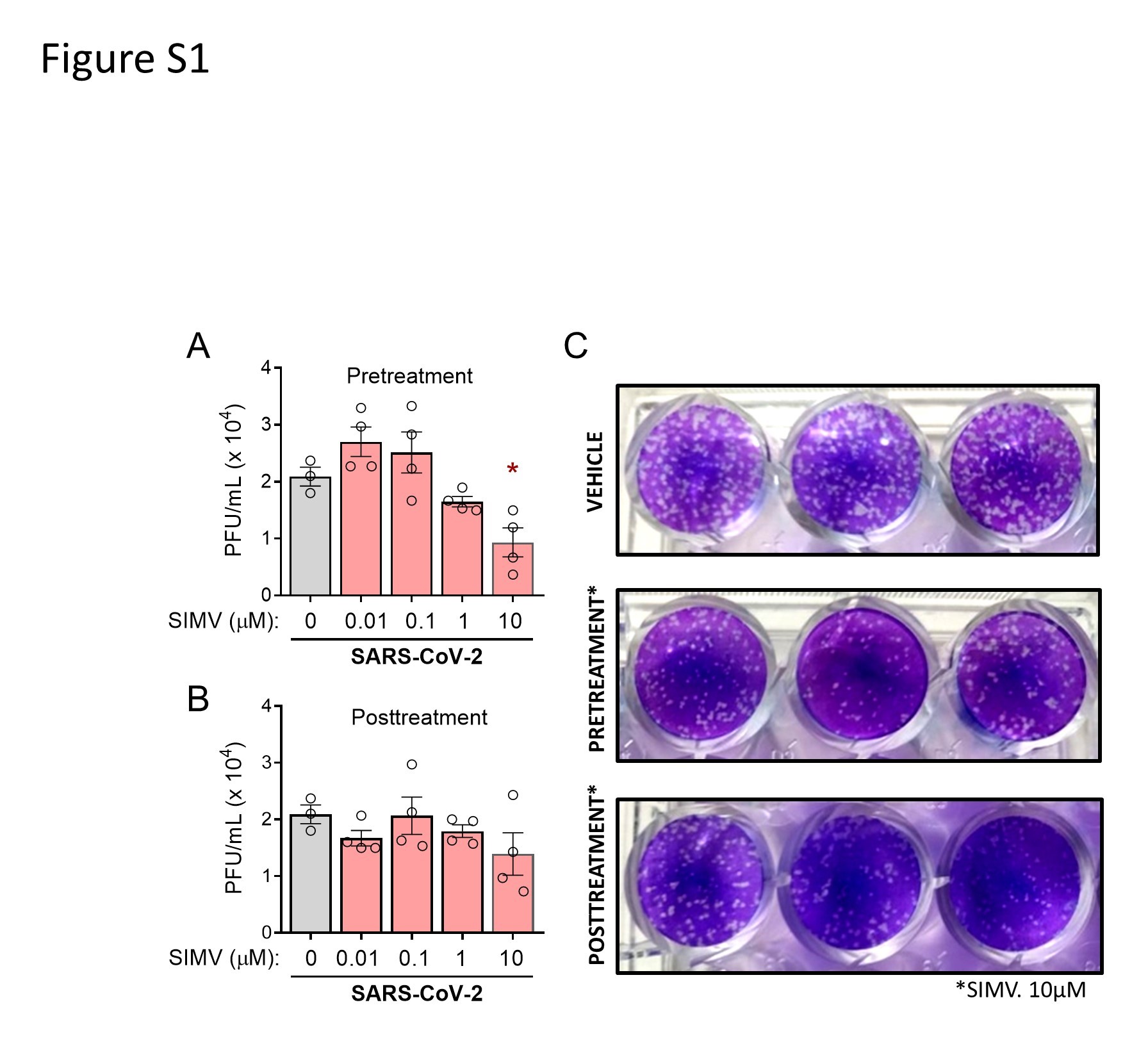

Supplement: Supplementary file 1 [file Image_1.jpeg]

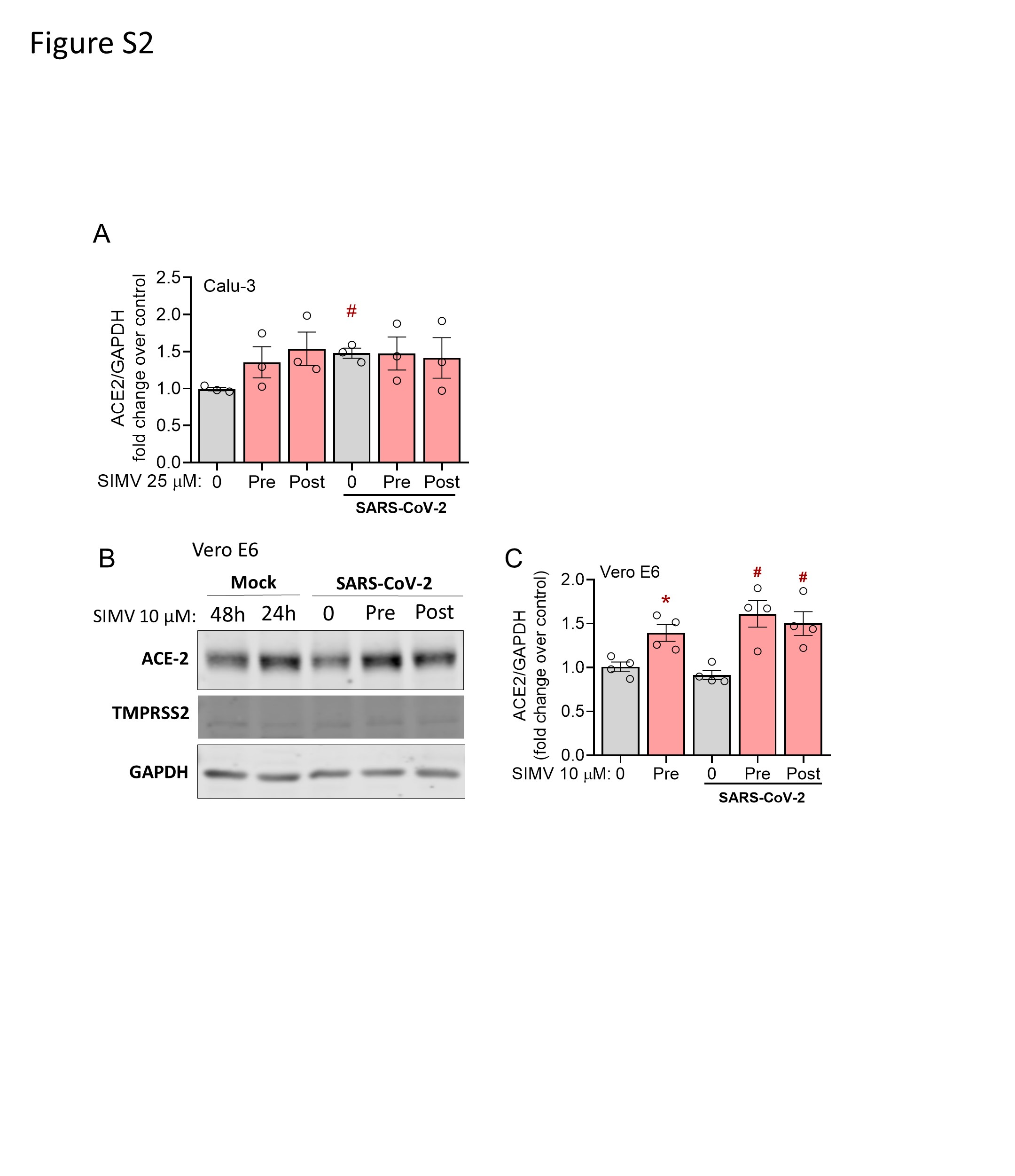

Supplement: Supplementary file 2 [file Image_2.jpeg]
